# Genome and transcriptome analysis to understand the role diversification of cytochrome P450 gene under excess nitrogen treatment

**DOI:** 10.1186/s12870-021-03224-x

**Published:** 2021-10-06

**Authors:** Rui Xiong, Ting He, Yamei Wang, Shifan Liu, Yameng Gao, Hanwei Yan, Yan Xiang

**Affiliations:** 1 Laboratory of Modern Biotechnology, School of Forestry and Landscape Architecture, Anhui Agricultural University, Hefei, 230036 China; 2 National Engineering Laboratory of Crop Stress Resistance Breeding, Anhui Agricultural University, Hefei, 230036 China

**Keywords:** Cytochrome P450, *Panax notoginseng*, Gene duplication, Expression profile, Nitrogen treatment

## Abstract

**Background:**

*Panax notoginseng* (Burk.) F. H. Chen (*P. notoginseng*) is a medicinal plant. Cytochrome P450 (CYP450) monooxygenase superfamily is involved in the synthesis of a variety of plant hormones. Studies have shown that CYP450 is involved in the synthesis of saponins, which are the main medicinal component of *P. notoginseng*. To date, the *P. notoginseng* CYP450 family has not been systematically studied, and its gene functions remain unclear.

**Results:**

In this study, a total of 188 *PnCYP* genes were identified, these genes were divided into 41 subfamilies and clustered into 9 clans. Moreover, we identified 40 paralogous pairs, of which only two had Ka/Ks ratio greater than 1, demonstrating that most *PnCYPs* underwent purification selection during evolution. In chromosome mapping and gene replication analysis, 8 tandem duplication and 11 segmental duplication events demonstrated that *PnCYP* genes were continuously replicating during their evolution. Gene ontology (GO) analysis annotated the functions of 188 *PnCYPs* into 21 functional subclasses, suggesting the functional diversity of these gene families. Functional divergence analyzed the members of the three primitive branches of CYP51, CYP74 and CYP97 at the amino acid level, and found some critical amino acid sites. The expression pattern of *PnCYP450* related to nitrogen treatment was studied using transcriptome sequencing data, 10 genes were significantly up-regulated and 37 genes were significantly down-regulated. Combined with transcriptome sequencing analysis, five potential functional genes were screened. Quantitative real-time PCR (qRT-PCR) indicated that these five genes were responded to methyl jasmonate (MEJA) and abscisic acid (ABA) treatment.

**Conclusions:**

These results provide a valuable basis for comprehending the classification and biological functions of *PnCYPs*, and offer clues to study their biological functions in response to nitrogen treatment.

**Supplementary Information:**

The online version contains supplementary material available at 10.1186/s12870-021-03224-x.

## Background

*Panax notoginseng* (Burk.) F. H. Chen (*P. notoginseng*) belongs to the Araliaceae genus, and it is widely cultivated in Yunan province, China. *P. notoginseng* is a medicinal plant, and its medicinal records can be traced to 3000 years ago [1]. Previous researches have shown that *P. notoginseng* has numerous bioactive compounds, such as saponins, which are its the main medicinal ingredient [2]. A total of 56 dammarane-type saponins, such as ginsenoside Rg1, Rb1 and notoginsenoside R1, have been identified and characterized [3]. These compounds can be used to treat cardiovascular diseases and trauma [1]; promote immunoregulation [4], hepatoprotection [5]; and anti-carcinogenesis [6]. No oleanane-type saponins were found in *P. notoginseng*. This was the main difference in chemical composition between *P. notoginseng* and other ginseng plants [2]. Significantly, the cytochrome P450 (CYP450) genes play roles in both saponin synthesis and plant hormone synthesis.

The *CYP* gene family is large and intricate, and is involved in the synthesis of various primary and secondary metabolites, such as phenylpropanes, terpenoids, alkaloids, fatty acids and hormone precursors [7]. *CYP* genes are widely found in animals, plants, fungi and bacteria [8]. Those genes are usually divided into 10 clans, including six single clans (clan 51, clan 74, clan 97, clan 710, clan 711, and clan 727) and four multiple gene family clans (clan 71, clan 72, clan 85, and clan 86) [9]. The functions of some CYP450 subfamilies have been elucidated. CYP74B, CYP703 and CYP704 can catalyze the production of fatty acid hydroxylase [10–12]. CYP74A, which also known as allene oxide synthase (AOS), is a key enzyme in jasmonate (JA) synthesis [13]. CYP97 is involved in abscisic acid (ABA) synthesis and participates in lutein synthesis [14]. In addition, CYP707A, which is present as ABA 8′-hydroxylases (ABA8ox) can catalyze phaseic acid synthesis. In gibberellin (GA) production, ent-kaurene oxidase (KO, CYP701A) catalyzes ent-kaurenoic acid formation [15]. Ent-kaurenoic acid oxidase (KAO) (CYP88A) uses the acid as a substrate to produce the GA12-aldehyde [16]. CYP90B, CYP90C, CYP90D, CYP72, CYP85A, CYP734A and CYP724B are responsible for brassinosteroid (BR) biosynthesis [7]. CYP71 enzyme in Sorghum is known to participate in the biosynthesis of the benzoquinone allelochemical sorgoleone [17]. Furthermore, the two nicotine n-demethylase genes (CYP82E) destroy the formation of nicotine in tobacco [18]. Importantly, CYP450 is associated with saponin synthesis in some plants. *Avena spp.* CYP51H10 [19], *Glycine max* (L.) Merr. CYP93E1 [20], CYP88D6 and CYP72A154 of *Glycyrrhiza uralensis* Fisch [21, 22]., CYP716A12 in *Medicago truncatula* L. have been reported to be involved in the synthesis of saponin [23].

Apart from the function on metabolites synthesis, some *CYP450* genes also play roles in resistance to stresses, such as drought, salt, pest infestation and bacterial pathogens. For instance, *OsDSS1*, which is a member of the cytochrome CY450 gene family, and its mutants (*dwarf and small seed 1*, *dss1*) enhance *Oryza sativa* drought tolerance due to the accumulation of ABA and metabolites [24]. The *AtCYP709B3* T-DNA insertion mutants (*cyp709b3*) show a salt intolerance phenotype [25]. The transient expression of saponin synthesis pathway genes in tobacco leaves indicates that *BvCYP72A552* catalyzed the formation of hederagenin-based saponins [26]. The reaction products of hydroperoxide lyase (HPL, CYP74B) in *Arabidopsis thaliana*, can be involved in the deterrence of insect pests [27]. Oscyp71Z2 overexpression plants show obvious resistance to bacterial fusarium caused by rice-XOO [28]. Some *CYP450* genes have a metabolic detoxification function, and are involved in the decomposition of environment toxins [29]. Under treatment with basic macronutrients such as nitrogen, the expression level of some genes of *CYP450* can also change. Decreased transcript levels of *PtCYP711A1* have been detected in roots with excess N treatment, and the transcript level of *PtCYP707A1* is up-regulated in N-starved roots [30]. In addition to its functions on compounds synthesis and biological or abiotic stress resistance, CYP450 has also been reported to be a growth factor. The CYP78A mutation in Arabidopsis and rice indicate that CYP78A is involved in regulating organ size and cell proliferation [7].

In recent years, many articles focused on the *CYP* gene family systematical studies were published, including *A. thaliana* [31], *O. sativa* [32], *Vitis vinifera* [33] and *Panax ginseng* [34]. Whereas, identification, structural and evolutionary analysis of the *PnCYP* gene family have not been reported, and there are few studies on the function of *PnCYP* genes. In this study, we performedgenome-wide bioinformatics analysis of the *PnCYP* gene family, including phylogenetic analysis, gene structure, conserved motifs, chromosomal locations, gene duplications, cis-regulatory elements and functional divergence. Meanwhile, due to the high medicinal value of *P. notoginseng*, abusing nitrogen (N) fertilizer is very common in *P. notoginseng* planting [35, 36]. Previous studies have shown that plant hormones combined with N signals regulate root growth and development [30, 37, 38], thereby the *PnCYPs* expression pattern under nitrogen treatment have been studied. Genes related to the synthesis of plant hormones (ABA, MEJA, GA) and saponins were screened by transcriptomic data analysis, the tissue expression pattern and change trend of gene expression levels under hormone treatment were analyzed by quantitative real-time PCR (qRT-PCR). Furthermore, some studies have predicted that *CYP* genes may be the miRNA target [32, 39], so we utilized this prediction in our paper. All of these were helpful to study the CYPs function of *P. notoginseng*, and optimize the plant environment of *P. notoginseng* to cultivate excellent varieties.

## Results

### Classification of identified PnCYP450s

The 188 putative *CYP450* genes were obtained from *P. notoginseng*. Here, we designated the *PnCYP* genes according to the classification Of*p. ginseng CYP* genes by sequence similarity. The genes were classified into 9 clans with 40 subfamilies. They were grouped into A-type (CYP71 clan) with 17 subfamilies comprising 102 genes and non-A type (CYP51, 72, 74, 85, 86, 97, 710, 711 clans) with 18 subfamilies including 86 PnCYPs. The CYP92, CYP703A, CYP710A, CYP711A family contained only one gene, while the CYP71 family was the largest family containing 27 memebers. The sequence length of 188 CYP450 genes ranged from 618 to 6279 bp and the molecular weights (Mw) of these proteins ranged from 22.5 to 239 kDa. The isoelectric points ranged from 5.17 to 9.51 (Table S1).

### Phylogenetic analyses and selective pressure analyses of PnCYP genes

**Fig. 1:**
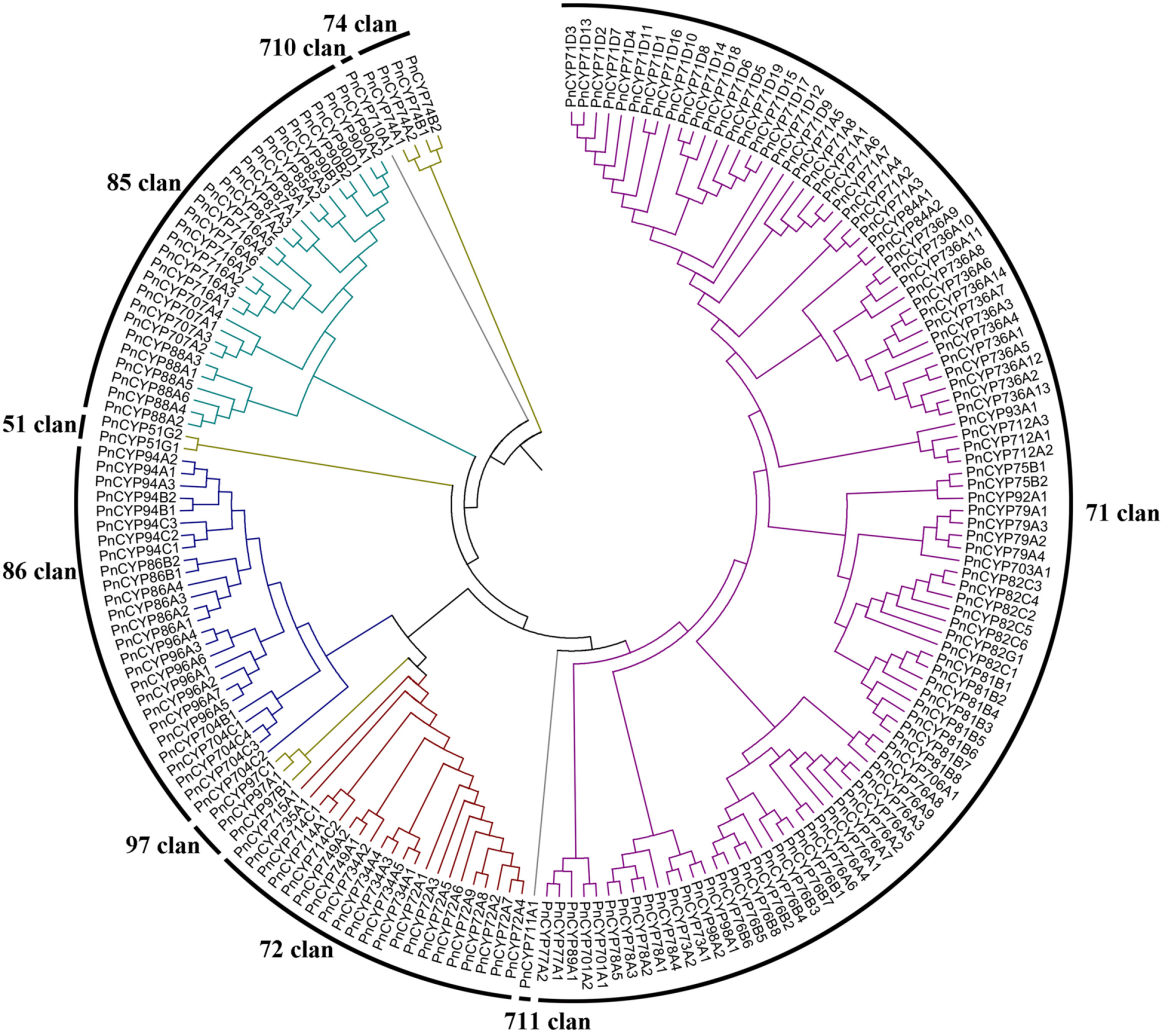
Phylogeny of the PgCYP gene superfamily. The unrooted phylogenetic tree of 187 PnCYP genes was constructed by MEGA5.0 with the Neighbor-Joining (NJ) method. Details of the CYP450 genes from Panax notoginseng are listed in Table S1

An unrooted neighbor-joining (NJ) phylogenetic tree of 188 *P. notoginseng* genes was built to explore the relationships among *PnCYP* genes. Those genes were classified into nine clans, some clans (CYP51, CYP74, CYP97, CYP710, CYP711) contained only one gene family, while the others (CYP71, CYP72, CYP85, CYP86) were multifamily clans (Fig. 1). CYP71 was the largest CYP450 clan with functional diversity. In *P. notoginseng*, the members of this branch accounted for 54.3% of the total. CYP71 clan members in other plants have been reported to play roles in plant secondary metabolism, such as amino acid derivatives, fatty acids, alkaloids, terpenoids and precursors of hormones [9].

To investigate the evolutionary relationships of plant CYP450s, an unrooted NJ tree among *P. notoginseng*, *A. thaliana*, *P. ginseng*, *O. sativa*, *P. trichocarpa* and *P. patens* was constructed (Fig. S1). A total of 47 subfamilies were identified in Arabidopsis, of which seven subfamilies (CYP73, CYP83, CYP93, CYP702, CYP705, CYP708, CYP709) were not found in *P. notoginseng*. Forty-one subfamilies were identified in *P. ginseng*, two subfamilies (CYP720 and CYP724) were not found in *P. notoginseng*, while CYP710 was not found in ginseng. Some families were only found in moss, including CYP752, CYP753, CYP751, CYP762 and CYP764 families*.* Meanwhile, CYP99 family, CYP723 family and CYP729 family were only present in rice*.* Moreover*, O. sativa* contained the CYP51H and CYP51G subfamilies, and other species only contained the CYP51G subfamily. *A. thaliana*, *P. notoginseng*, *P. ginseng* contained only the CYP74A and CYP74B subfamilies, but the other three plants contained at least three CYP74 subfamilies (e.g. CYP74C, CYP74D et al.).

To further explore the evolutionary patterns of the *CYP450* gene family in plants, paralogs of *P. notoginseng*, orthologs betweeen *P. notoginseng* and *A. thaliana*, *P. notoginseng* and *P. ginseng* were identified and listed in Table S2. Forty paralog pairs in Pn-Pn, 26 ortholog pairs in Pn-At, 70 ortholog pairs in Pn-Pg were identified (Table S2). To investigate the selection pressure of the PnCYP450 genes, the non-synonymous substitution rate/ synonymous substitution rate (Ka/Ks) value of these homologous pairs was calculated (Table S3 and Table S4). In total, only two paralog pairs (*PnCYP736A2/PnCYP736A12*, *PnCYP716A2/PnCYP716A7*) in Pn-Pn had Ka/Ks ratios greater than 1. Six orthologs pairs (*PnCYP76A1/PgCYP76A2v2, PnCYP81B2/PgCYP81D3p*, *PnCYP72A4/PgCYP72A8*, *PnCYP74B1/PgCYP74B1*, *PnCYP707A1/PgCYP707A3*, *PnCYP704C2/PgCYP704C4*) had Ka/Ks ratios larger than 1 (Fig. S2). All Ka/Ks values of *Pn-At* orthologs pairs were less than 1. This indicated that most genes have undergone purification selection.

### Gene structure and duplication analyses

**Fig. 2:**
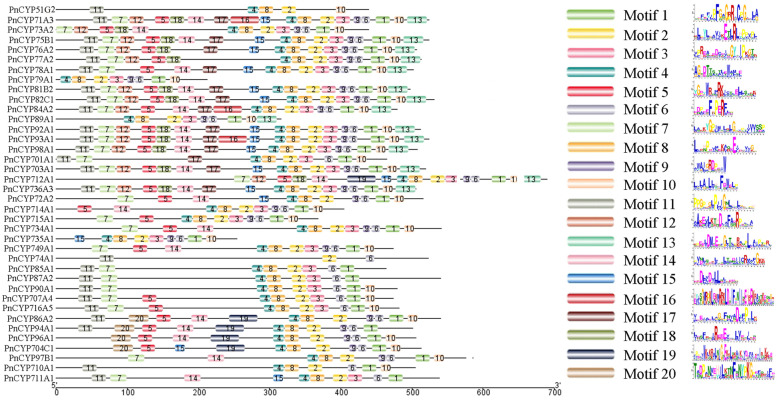
The conserved motifs of PnCYP genes and their distribution in the genes. Conserved motifs in PnCYP genes were identified using MEME. Different colored boxes represent different motifs. Box lengths in the figure do not represent actual relative motif sizes

The conserved motifs of CYP450 proteins in *P. notoginseng* were searched (Fig. 2 and S3). Some motifs were presented in most proteins, such as the K-helix (motif 2, EXXR), and motif 6 (proton-transfer groove (PERF)). Motifs 4 and 8 existed in almost all families except the CYP74 clan. While some motifs were only presented in specific families, such as motifs 12, 16 and 17 in the CYP71 clan, motif 20 in CYP86 clan. Obviously, members of CYP51 clan and CYP74 clan appeared to lose some motifs. The motif 11–7–12-5-18-14-17-15-4-8-2-3-9-6-1-10-13 layout was generally conserved in the CYP71 clan, motif 1 (CXG) and motif 4 (AGXD/ET) were related to functional definition. The motif layout (11–20–5-14-19-4-8-2-9-6-1) was universally conserved in CYP86 clan. Motif 11–4–8-2-10 layout in CYP51 clan and motif 11–7–4-8-2-3-6-10 layout in the CYP85 clan.

We analyzed the gene structure of the *PnCYP* genes (Fig. S3). Some subfamilies consisted of intronless genes, such as CYP77 and CYP710. In 71 clan, most members (83 genes, 81.4%) contained only one intron, while all CYP701 subfamily members contained six introns. The characteristic of 72 clan was that most genes had 4 introns. In 86 clan, almost all subfamilies (CYP86, CYP94 and CYP96) members had only one intron except the CYP704 subfamily. In contrast, CYP97 clan and CYP85 clan had very complex gene structures, *PnCYP97A1* contained the maximum number of 15 introns.

**Fig. 3:**
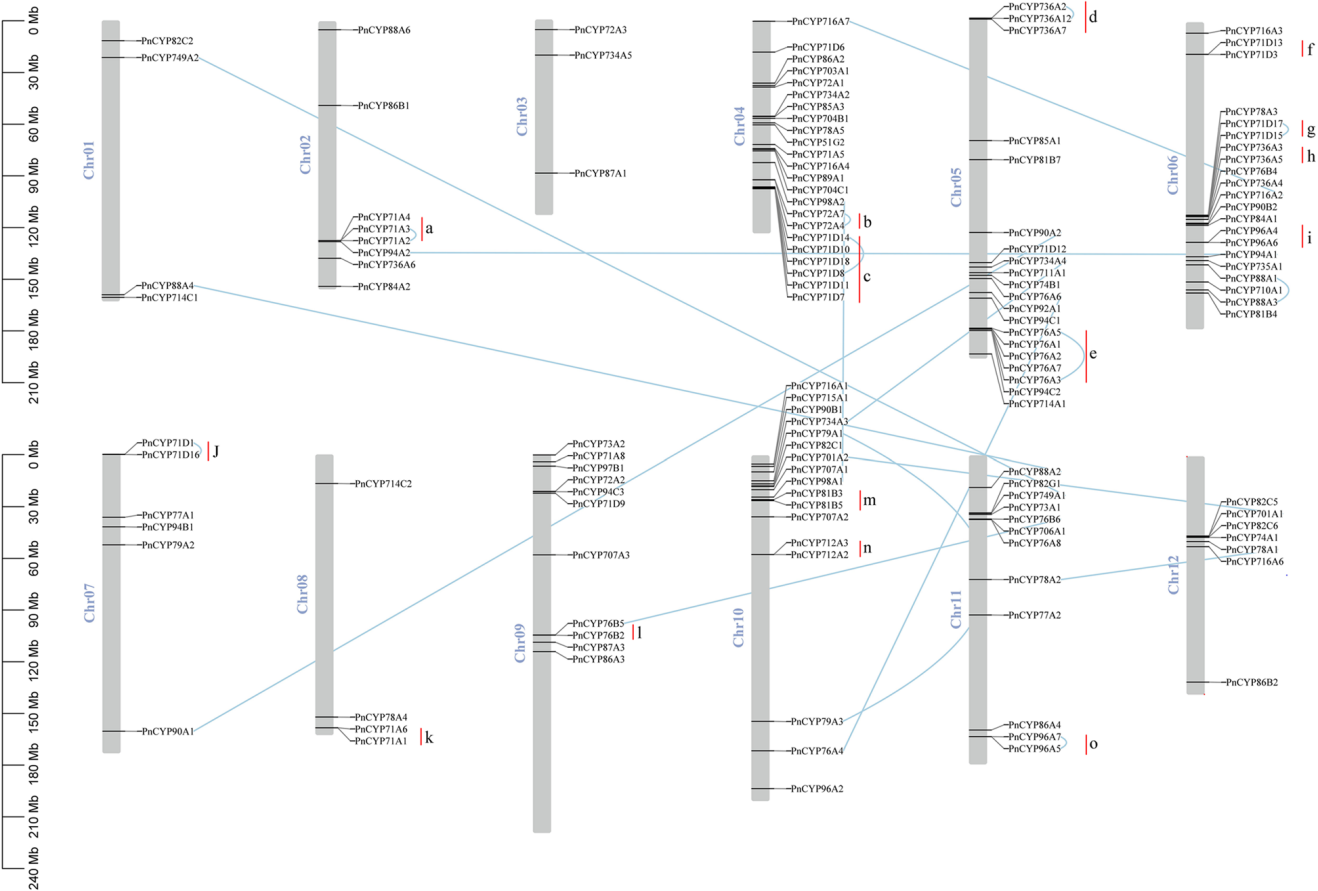
Chromosomal locations of PnCYP genes. The 137 SCPL genes are widely mapped to all of the 12 *Panax notoginseng* chromosomes. Chromosome numbers are indicated at the top of each bar, gene names on the left of each bar corresponding to the approximate locations of each gene member. The duplicated paralogous pairs of PnCYP genes are connected with blue lines, the genes within metabolic gene clusters are marked with red line and black characters (a,b,c etc.)

A total of 136 (72.7%) *PnCYP450* genes were unevenly localized on the 12 chromosomes (Fig. 3). Chromosome 3 had the least genes at three, and chromosome 4 contained the most genes (23 total). Relatively higher gene density was observed in chromosome 4, 5, 6 and 10 regions. We defined 15 gene clusters (labelled a-o), the largest cluster (cluster e) which composed of 6 members was found on chromosome 5, and 8 paralogous pairs were found and thought to be evolved in tandem duplication (*PnCYP71A2*/*PnCYP71A2*, *PnCYP72A4*/*PnCYP72A7*, *PnCYP71D8*/*PnCYP71D14*, *PnCYP736A2*/*PnCYP736A12*, *PnCYP76A3*/*PnCYP76A5*, *PnCYP71D15*/*PnCYP71D17*, *PnCYP71D1/PnCYP71D16*, *PnCYP96A5*/*PnCYP96A7*). And 11 segmental duplication events were detected in total.

### Cis-regulatory elements analyses and miRNA target prediction

**Fig. 4:**
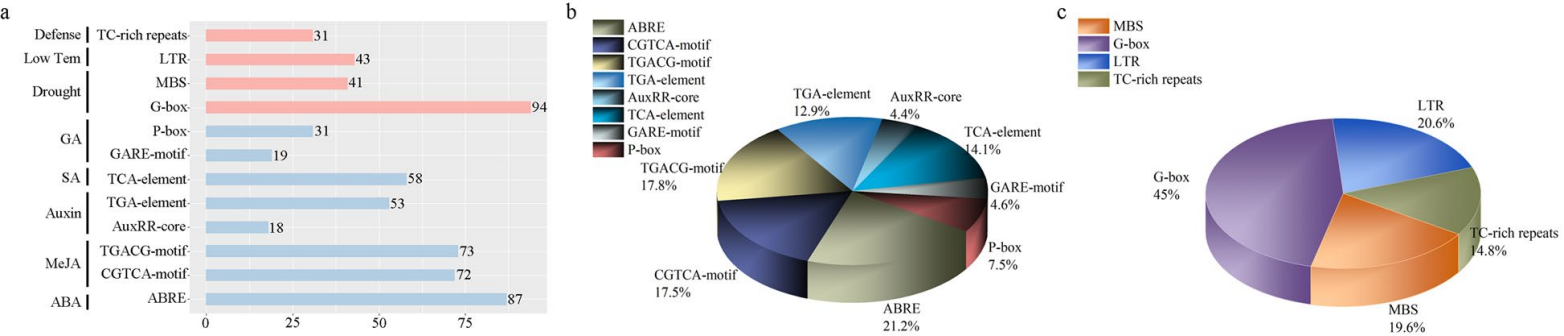
Cis-acting element analysis of the promoter regions of PnCYP genes. Based on functional annotation data, cis-acting elements were classified into two major classes: phytohormone responsive elements (i.e. those responsive to ABA, auxin, GA, MeJA, and/or SA) and abiotic stress response cis-acting elements (e.g. those involved in plant defense, drought stress response, and/or low temperature stress response). **a** Number of each cis-acting elements in the promoter regions of PnCYP genes. **b** Percentage of total cis-acting elements in the promoter region of the PnCYP gene. **c** and **d** The number of each cis-acting element as a percentage of its classification

The 136 gene promoters contained two kinds of cis-regulatory elements (Fig. 4, Table S5), one of which was related to biological stress. Among these cis-regulatory regulatory elements, ABA response element (ABRE) accounted for the maximum ratio, 87 gene promoters (64%) contained this element. The cis-regulatory elements associated with MEJA (CGTCA-motif and TGACG-motif) were respectively found in 72 and 73 gene promoters. There were another two cis-regulatory elements with a higher proportion: TGA-element (IAA-related, 53 genes) and TCA-element (salicylic acid (SA)-related, 58 genes). The promoters related to biological stress also included the GARE-motif and P-box elements which are involved in GA response. The other cis-regulatory element was related to abiotic stress, including MBS (41 genes) and G-box (94 genes) elements related to drought, low temperature stress response (LTR, 43 genes) and TC-rich (31 genes) repeats related to defense.

Three pairs of miRNA-target interactions were found, containing 2 miRNAs and 3 CYP450, (Table S6). MiR164 mediated transcriptional regulation of PnCYP736A9 and PnCYP736A10, while PnCYP86A3 was possibly a target of miR171. Both MiR164 and miR171 are associated with stress resistance in other species [40], while PnCYP736A9 and PnCYP86A3 were responded to nitrogen treatment in this study.

### GO, Kyoto encyclopedia of genomes (KEGG) annotation and subcellular localization prediction of the PnCYP450 gene superfamily

**Fig. 5:**
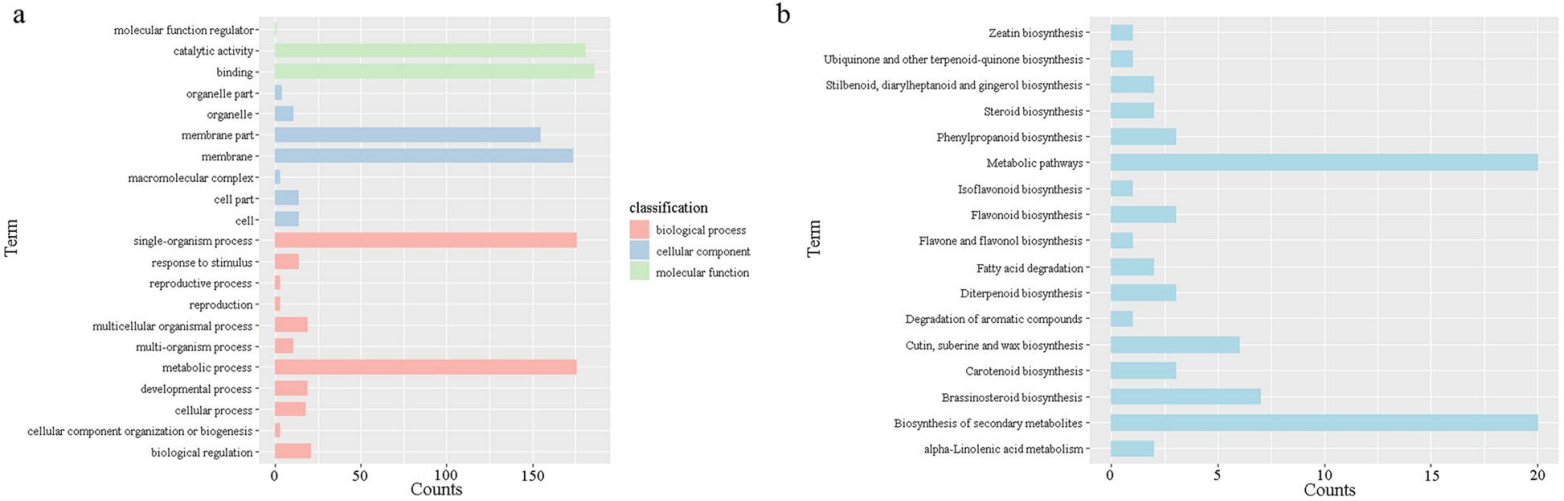
Annotation analysis of PnCYP proteins. **a**: Gene ontology (GO) annotation of PnCYP proteins. Bars indicate the number of genes with the same term. **b**:Kyoto Encyclopedia of Genes and Genomes (KEGG) annotation of PnCYP proteins

A total of 187 PnCYP450s were annotated on Blast2GO software except *PnCYP715A1*. Those CYP450 protein were assigned to 21 GO terms (Fig. 5a and Table S7). The terms were classified into three major functional categories, including 11 biological process subcategories, 7 cellular component subcategories and 3 molecular function subcategories. A total of 174, 176, and 186 proteins were annotated on the terms of biological process, cellular component, and biological process, respectively. Most genes were associated with metabolic reactions under biological process (GO:0008152), indicating that CYP450 genes function in the synthesis of various primary and secondary metabolites. Only three genes (PnCYP703A1, PnCYP90A2, PnCYP90A1) were predicted to be involved in cellular component organization or biogenesis (GO:0071840, GO:0022414, GO:0000003). For cellular component, most genes were related to membrane part (GO:0016020, GO:0044425) and only three genes (PnCYP94A1, PnCYP94A2, PnCYP86B1) were related to macromolecular complex (GO:0032991). On the terms of molecular function, most genes were shown to play roles in catalytic process (GO:0003824) but only one gene (PnCYP94A3) was related to the molecular function regulator.

A total of 77 PnCYP450s were annotated in the KEGG database. These genes were annotated on 17 pathways which were most related to compound synthesis (Fig. 5b). PnCYP73A1 and PnCYP73A2 were annotated into degradation of aromatic compounds (ko01220). PnCYP74 subfamily was related to alpha-Linolenic acid metabolism (ko00592). PnCYP82, PnCYP88, PnCYP701 subfamilies were shown to play roles in diterpenoid biosynthesis (ko00904). And PnCYP97, PnCYP707 subfamilies were predicted to have a function on carotenoid biosynthesis (ko00906). This indicates that CYP450 genes from different subfamilies may play roles in the same metabolic pathway.

The subcellular localization analysis of the 188 PebHLH proteins predicted they were mostly located in the cytoplasmic (114, 60.6%), 65 members were located in innermembrance (34.6%), six were located in outermembrance (3.2%), only three were located in periplasmic (1.6%) (Table S7).

### Functional divergence analysis of the CYP51, CYP74 and CYP97 clans

We calculated the Type I and Type II functional divergence correlation coefficient of CYP51, CYP74, and CYP97 clans, because these clans were conserved throughout evolution and had different functions [39]. As shown in Table 1 and S8, those three families were divided into five clusters: CYP51H/CYP51G, CYP74A/CYP74B, CYP97A/CYP97B, CYP97A/CYP97C, CYP97B/CYP97C. In CYP51H/CYP51G pairs, Type I functional divergence coefficient was significantly greater than 0 (θ_I_, 0.4729 ± 0.135917, *P < 0.05), a total of 14 critical amino acid sites (CAASs) were detected. But the Type-II functional divergence coefficients (θ_II_) of this pair was less than 0. A similar situation was also found in CYP74A/CYP74B pairs, the Type I functional divergence coefficient was statistically significant (θ_I_, 0.2984 ± 0.091536, *P < 0.05) and contained six CAASs. However, Type-II functional divergence coefficient was negative. These findings indicated that evolution rate divergence and evolution property divergence of CYP51, CYP74 clan were inconsistent. Meanwhile, the Type-I and Type-II coefficients of CYP97A/CYP97B pair (θ_I_, 0.5808 ± 0.187075, *P < 0.05; θ_II_, 0.2443 ± 0.058375, *P < 0.05), CYP97A/CYP97C pairs (θ_I_, 0.5336 ± 0.161246, *P < 0.05; θ_II_, 0.2374 ± 0.055073, *P < 0.05), CYP97B/CYP97C pairs (θ_I_, 0.4912 ± 0.150662, *P < 0.05; θ_II_, 0.2409 ± 0.058073, *P < 0.05) were all significant. In Type I fuctional divergence, there were 29, 24, 17 CAASs in CYP97A/CYP97B, CYP97A/CYP97C, CYP97B/CYP97C clusters, respectively. While in Type-II functional divergence, 52, 62 and 69 CAAs were detected in in CYP97A/CYP97B, CYP97A/CYP97C, CYP97B/CYP97C clusters, respectively.

**Table 1:** Site-specific profiles for two types of functional divergence (Type-I and Type-II), measured by the posterior ratio

Comparison pairwise	Types	θ	LRT	***p***-value	Cutoff of posterior probability	Number of RFD
CYP51H/CYP51G	I	0.4729 ± 0.135917	12.105816	< 0.01	0.694309	14
CYP74A/CYP74B	I	0.2984 ± 0.091536	10.627121	< 0.01	0.671592	6
CYP97A/CYP97B	I	0.5808 ± 0.187075	9.638727	< 0.01	0.684058	29
CYP97A/CYP97C	I	0.5336 ± 0.161246	10.951024	< 0.01	0.673728	24
CYP97B/CYP97C	I	0.4912 ± 0.150662	10.629417	< 0.01	0.701093	17
CYP97A/CYP97B	II	0.2443 ± 0.058375		< 0.01	1.126562	52
CYP97A/CYP97C	II	0.2374 ± 0.055073		< 0.01	1.076256	62
CYP97B/CYP97C	II	0.2409 ± 0.058073		< 0.01	1.151247	69

θ: the coefficients of Type-I and Type-II functional divergence between two gene clusters; *LRT* Likelihood ratio statistic

Cutoff of posterior probability: minimum posterior probability of amino acid sites leading to functional divergence

Number of RFD: the predicted number of amino acid sites associated with functional divergence

### Expression analyses of PnCYP450 genes treated with different forms of nitrogen fertilizers

**Fig. 6:**
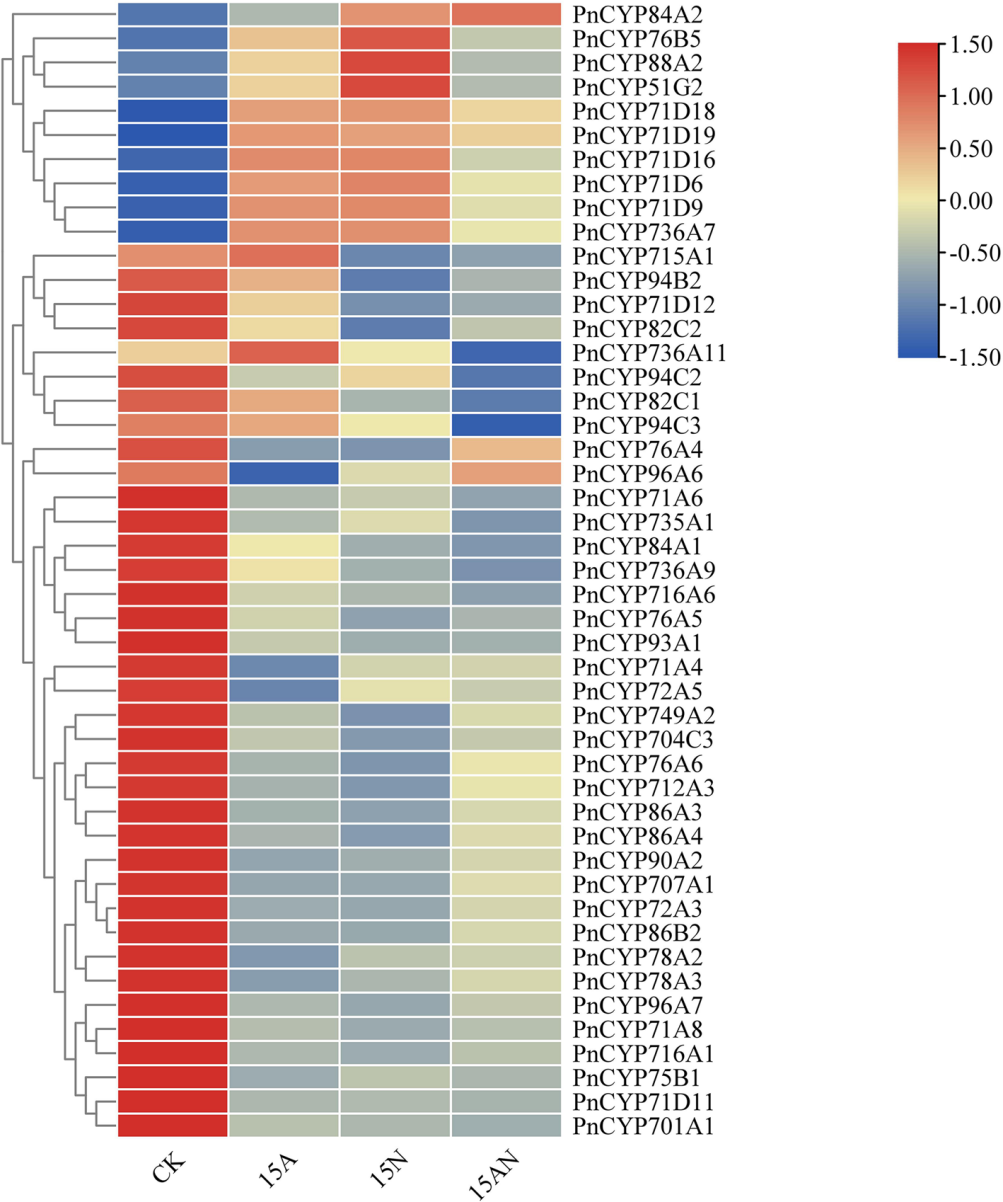
The expression profiling of PnCYPs through different nitrogen treatments. 15A: 15 mM NH4+; 15 N: 15 mM NO3−;15AN: 15 mM NH4++ 15 mM NO3−

Excessive use of ammonium salt as the sole source of nitrogen hindered plant growth. To analyze expression levels of CYP450s in response to nitrogen fertilizers treatment, 188 PnCYP genes expression data (Per Kilobase of exon model per Million mapped reads, FPKM) were counted (Table S9). One hundred seven PnCYP genes whose expression data > 1 in one or more treatment were selected for further analysis (Fig. S4, Table S10). A total of 47 differentially expressed genes were identified, among them, 10 genes were significantly up-regulated and 37 genes were significantly down-regulated (fold change (FC) > 1.5 and *p*-values < 0.05) (Fig. 6, Table S11). Five genes (PnCYP71D6, PnCYP71D9, PnCYP71D16, PnCYP76B5, PnCYP736A7) were significant up-regulated under 15A and 15 N treatment, however, when the plants were treated with 15AN, the expression levels of these genes did not change significantly. The expression values of four genes (PnCYP71D18, PnCYP71D19, PnCYP84A2, PnCYP88A2) were significantly increased under the three treatments. While the expression levels of PnCYP93A2, PnCYP704C3, PnCYP716A6, PnCYP86B2, PnCYP93A4, PnCYP734A6, PnCYP78A2, PnCYP76A5, PnCYP71A6, PnCYP96A7, PnCYP72A3, PnCYP75B1, PnCYP90A2, PnCYP71A4, PnCYP701A1, PnCYP707A1, PnCYP716A1, PnCYP71A8, PnCYP71D11 and PnCYP78A3 were reduced under all three treatments. PnCYP735A2, PnCYP82C1, PnCYP736A9, PnCYP71D12, PnCYP94B2 and PnCYP84A1 exhibited decreased expression under 15 N and 15AN treatments. PnCYP76A4, PnCYP76A6 and PnCYP86A4 responded to 15A and 15 N treatment. PnCYP94C3, PnCYP94C2, PnCYP735A5, PnCYP86A3, PnCYP72A5 and PnCYP96A6 only responded to one treatment.

### Quantitative real-time PCR (qRT-PCR) expression profiles of *PnCYP* genes

**Fig. 7:**
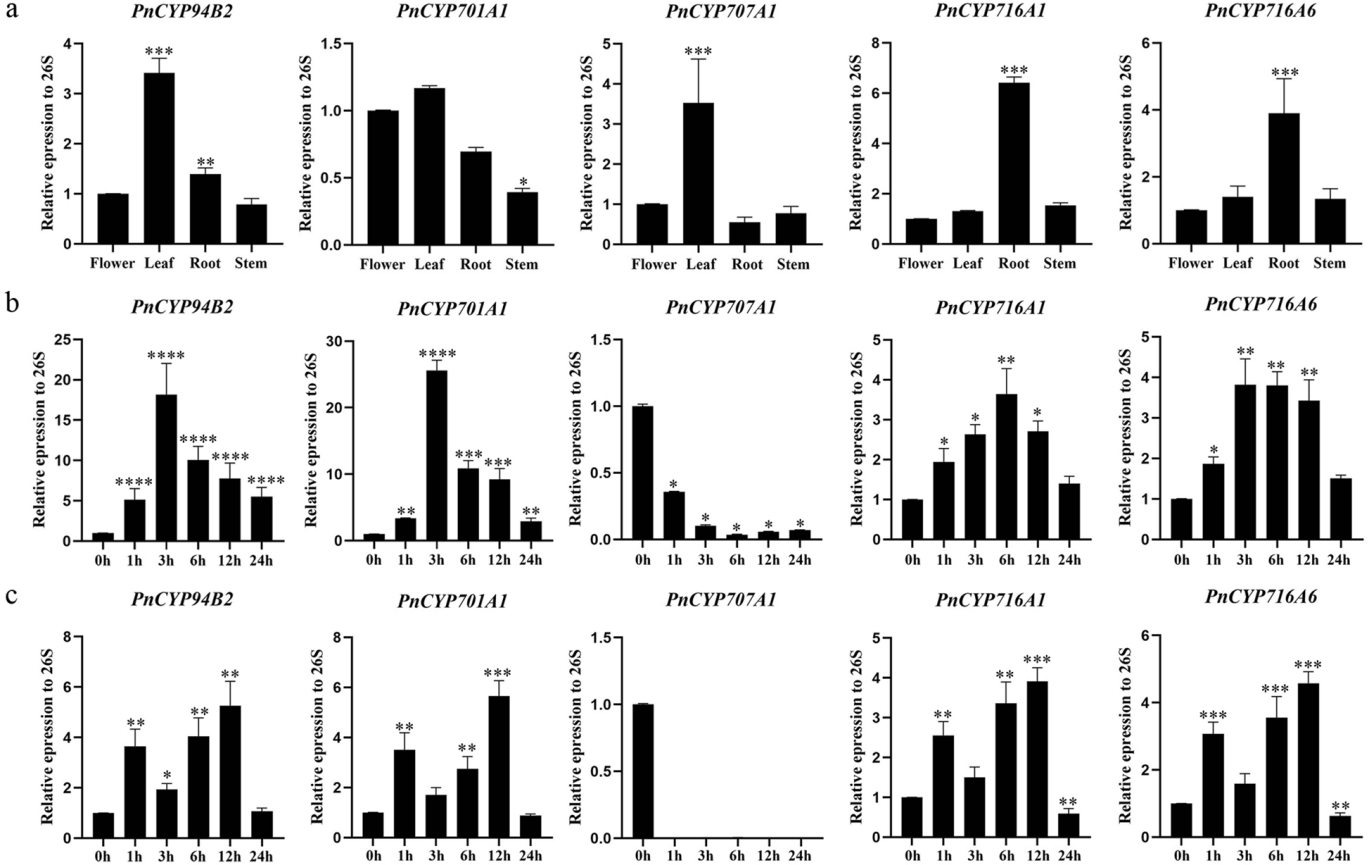
Expression patterns of selected PnCYP genes. **a**: Expression profiles of Panax bamboo CYP genes in different tissues. **b**: Expression analysis of 5 CYP genes in Panax notoginseng following ABA treatment as determined by qRT-PCR. **c**: Expression analysis of 5 CYP genes in Panax notoginseng following MEJA treatment as determined by qRT-PCR

Nitrogen treatment can affect the synthesis of some hormones (ABA, MEJA, GA) in plants. In the present study, a MEJA synthesis related gene (PnCYP94B2), one gene related to GA synthesis (PnCYP701A1) and one gene related to ABA synthesis (PnCYP707A1) were screened for the further study. The main medicinal components in *P. notoginseng* were saponins. Therefore, two saponin synthesis related genes (PnCYP716A1, PnCYP716A6) were screened. Primers used for the qRT-PCR analysis of PnCYP gene expression were listed in Table S13. Tissue expression pattern analysis showed that the expression levels of PnCYP94B2, PnCYP701A1 and PnCYP707A1 in leaves were higher than these in the other three tissues, while the expression levels of PnCYP716A1 and PnCYP716A6 in the roots were higher than these in the other three tissues (Fig. 7). We also analyzed the expression trends of these five candidate genes under ABA and MEJA treatment. The expression level of the PnCYP707A1 gene was significantly decreased under treatment with ABA and MEJA (> 2), while the expression levels of the remaining four genes were all increased under treatment with these two hormones. In the MEJA treatment, PnCYP94B2, PnCYP701A1, PnCYP716A1 and PnCYP716A6 showed similar changes, that is, the expression levels of PnCYP94B2, PnCYP701A1, PnCYP716A6 were significantly increased at 1 h, 6 h and 12 h (> 2). The expression patterns of PnCYP94B2 and PnCYP701A1 were similar in the ABA treatment, and the expression levels of PnCYP716A1 and PnCYP716A6 were significantly increased at 1 h, 3 h, 6 h, 12 h and 24 h (Fig. 7).

## Discussion

To investigate the evolutionary relationships of plant *CYP450s*, an unrooted NJ tree among *P. notoginseng*, *P. ginseng*, *Physcomitrella patens* (moss), *O. sativa* (rice), *A. thaliana* (Arabidopsis), *Populus trichocarpa* (poplar) was constructed (Fig. S1). The number of *PnCYPs* was less than that of Arabidopsis (245), poplar (310), rice (326), and more than algae (98) and ginseng (138) [31–34], indicating that the CYP family has a significant degree of evolutionary extension among plant species. The molecular weight (MW) and isoelectric point (PI) partly determined the gene molecular structure and biochemical function [41], and the large range of PnCYP MW and PI may be due to their role in different synthetic pathways, which also shows the functional diversity of PnCYP. Protein divergence analysis showed that even in the conservative subfamilies, functional divergence still existed. In 51, 74, and 97 subfamilies, the type I functional divergence coefficients (θ_I_) among all branches were greater than 0, indicating that functional divergence occurred between members of each subfamily. In addition, in the 97 subfamily, the θ_I_ coefficient was greater than that in the other two subfamilies, which proved that the functional divergence possibility in this subfamily was greater than that of 51, 74 subfamily. Identifying potential critical amino acid positions in CYP51, CYP74, and CYP97 proteins can provide more information for analyzing the evolution of the PnCYP450 family.

Some subfamilies and clans showed closer evolutionary relationship. For example, CYP83 and CYP99 family were inside the CYP71 family on the phylogenetic tree. The monocot specific CYP723 family was obviously closest to CYP89 family, CYP729 family was obviously closest to CYP88 family. CYP51 clan, CYP710 clan and CYP85 clan were gathered on the same branch. Some members of the three clans were reported to have function on sterol synthesis and processing. CYP51G is reported to be involved in the synthesis of sterols, some CYP710 family members are known as sterol C22-desaturases, CYP85 clan members can process plant sterols [42]. In summary, CYP710 clan and CYP85 clan may be evolved from CYP51. Meanwhile, different subfamilies in the CYP85 clan (CYP90 and CYP724B subfamilies) are involved in the synthesis of brassinosteroids [43, 44], and phylogenetic tree analysis showed that the two subfamilies were clustered together. CYP74, CYP727 and CYP751 were also clustered on the same branch. CYP751 was only presented in moss and was clustered on the clan of CYP727, so we predicted that 751 and 727 may come from the same ancestor. CYP97, CYP72 and CYP86 clustering together on the phylogenetic tree indicated that they may be evolved from a common ancestor. CYP97 contained three distinct subfamilies, and CYP97C was scattered in the CYP97B branch. This proved that in the evolutionary process, the genetic relationship between CYP97B and CYP97C was closer, compared with CYP97A. The members of CYP72 clan are relevant to the synthesis of plant hormones (GA), cytokinins, isoprenoids and fatty acids [11, 44]. Some members of CYP86 clan (CYP86 and CYP94 subfamilies) encode fatty acid hydroxylases or alkane hydroxylase [45, 46], and the two subfamilies were clustered together. Based on the above analysis, we speculated that the CYPS superfamily acquired new functions through duplicate divergence.

Motif and gene structure analysis strongly supported the results of subfamily classification. For some subfamilies in the same branch, motif compositions and gene structure were similar, such as the CYP71, CYP736 and CYP82 subfamilies in 71clan, indicating that these subfamilies might have similar functions. In this study, we found five cis-acting regulatory elements (ABA, MeJA, Auxin, SA and GA) associated with plant hormones were. The promoters of *PnCYP82C1*, *PnCYP701A1*, *PnCYP736A3* and *PnCYP714A1* contained all of these five cis-acting elements. At the same time, three cis-acting elements (Drought, Low Tem, Defense) associated with abiotic stress were found in the promoters. *PnCYP76A5*, *PnCYP76A6*, *PnCYP78A5* and *PnCYP72A2* contained all of these three cis-acting elements. This suggests that these genes may play an important role in the development of *P. notoginseng*. The expression patterns under nitrogen treatment revealed that only one paralog pair (*CYP76A4*/*CYP76A6*) had the similar expression pattern, suggesting functional divergence among PnCYP. Structural analysis showed that the frequency of segment duplication in *P. notoginseng* was greater than that of tandem duplication, and the Ka/Ks ratio of two paralogous pairs (Pn-Pn) and six orthologous pairs (Pn-Pg) was greater than 1.0. All of these demonstrated that in the process of evolution, some genes undergo positive selection, and the phenomenon of functional divergence did existed.

**Fig. 8:**
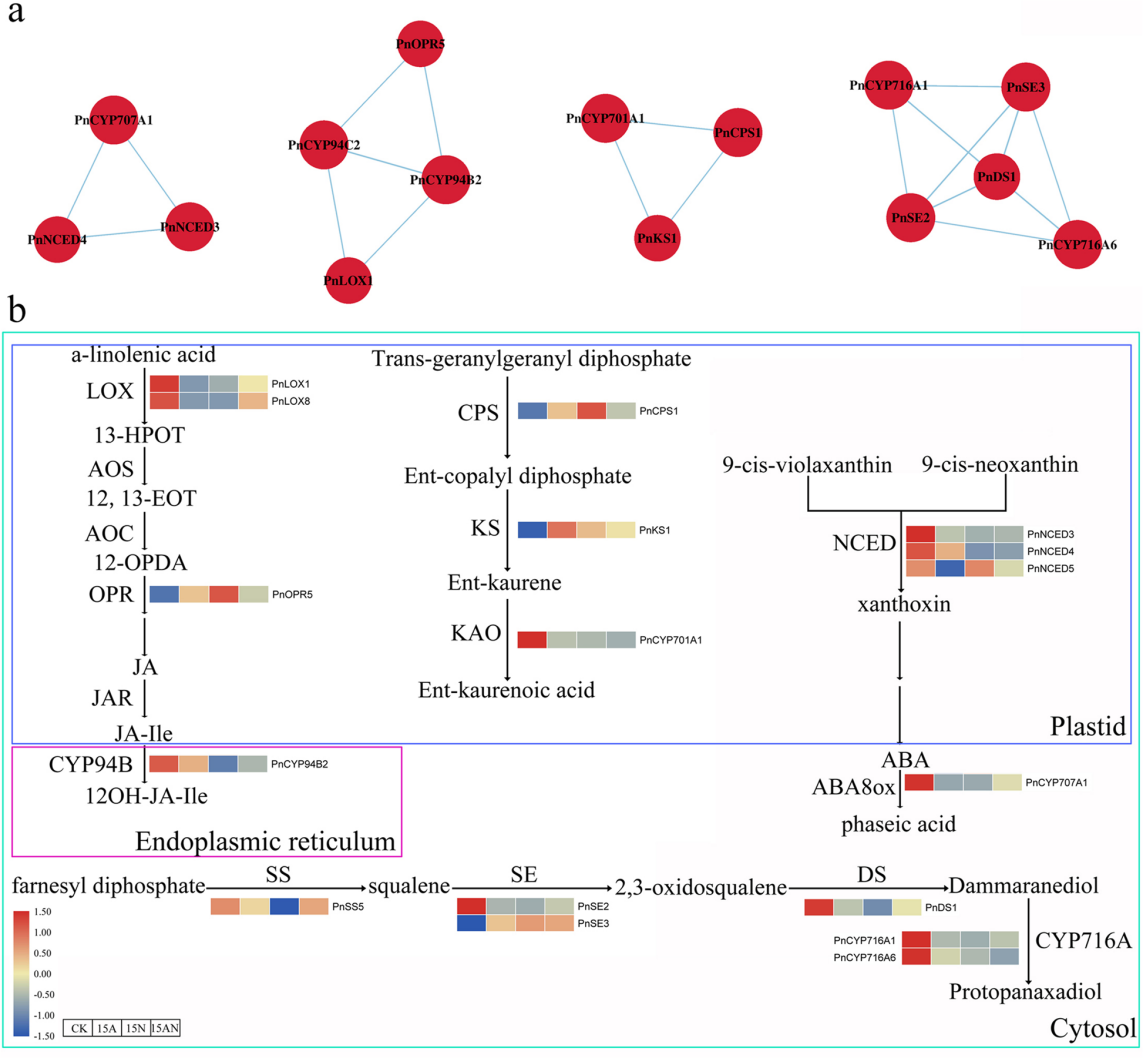
Analysis of the CYP450s expression under nitrogen treatment. **A**: Co-expression analysis of PnCYP450s involved in ABA, JA, GA, Saponins. **B**: A schematic diagram of CYP450-mediated signaling pathways under nitrogen treatment

In plants, nitrogen treatment affected some phytohormones’ synthesis, such as ABA, GA, JA and SA. Transcriptome data indicated that some genes related to hormone synthesis were responsive under the three conditions. CYP707A, which is also known as ABA8Ox, was incrementally down-regulated with the three kinds of treatments, *PnCYP707A1* showed significantly reduced expression levels with at least 2.3-fold. In the qRT-PCR experiment, the *PnCYP707A1* expression level was significantly decreased under ABA treatment (> 2) (Fig. 7). And *PnCYP707A1* promoter contained ABA relevant cis-element (ABRE). The 9-cisepoxycarotenoid dioxygenases (NCEDs) which were also associated with ABA synthesis were identified (Table S12). And *PnNCED3*, *PnNCED4* and *PnNCED5* showed sharp decrease of transcripts in at least one treatment. *PnNCED3* was down-regulated 4.4-fold and 6.2-fold respectively under 15A and 15AN treatment. While *PnNCED4* was significantly up-regulated in 15 N. Furthermore, *PnCYP707A1, PnNCED3* and *PnNCED4* showed a significant correlation of expression (Fig. 8a). In the JA synthesis pathway, two lipoxygenases (PnLOXs) (PnLOX1 and PnLOX8) were down regulated under 15A and 15 N treatment (Fig. 8b). Eight 12-oxophytodienoate reductases (*PnOPRs*) were identified, however, only one of them (*PnOPR8*) responded to nitrogen treatment. In Arabidopsis, *CYP94B1*, *CYP94B3* and *CYP94C1* were found to be associated with the partial deactivation of JA-Ile hormone [47]. *PnCYP94B2, PnCYP94C2* and *PnCYP94C3* whose promoters contain the CGTCA-motif and TGACG-motif (Table S5) were all differentially expressed genes in this study. In the qRT-PCR experiment, the *PnCYP94B2* expression level was significantly increased under ABA treatment (> 2) (Fig. 7). In co-expression network, *PnCYP94B2, PnCYP94C2* showed a connection to *PnLOX1* and *PnOPR8.* Moreover, GA synthesis is also affected by nitrogen in the soil [30]. Only one ent-copalyl diphosphate synthase gene (CPS) was identified and it (*PnCPS1*) had a more than 3-fold increase in expression within 15 N treatment. Its downstream gene, kaurene synthases enzyme (*PnKS1*) was up-regulated 2.2-fold when treated with 15A. And *PnCYP701A1* which was also involved in gibberellin synthesis and contained the GARE-motif in its promoter, showed significant downregulation in all treatments. Likewise, the co-expression analysis indicated that these three genes were closely related to one another (Fig. 8a).

Saponins are the main medicinal ingredients of *P. notoginseng* [2], and *CYP* genes participate in saponin synthesis [19–23], so the genes related to the saponin synthesis pathway were identified and their transcripts levels were counted. Squalene synthase (SS) was responsible for the synthesis of triterpene essential substrate (C30 isoprenoid squalene), which showed decreased expression levels under 15A (Fig. 8b). Triterpenoid compounds oxidation genes (squalene epoxidase [SE]) *PnSE2* showed at least 2.9-fold down regulation under all three conditions, while *PnSE3* was up-regulated at least 2.0-fold under all three conditions. Only one dammarenediol-II synthase (DS) gene, which was a kind of oxidosqualene cyclase, was found in *P. notoginseng*. It was down-regulated in response to 15A and 15 N. CYP716A, which plays roles in processing protopanoxadiol [23],also responded to nitrogen treatment. *PnCYP716A1* and *PnCYP716A6* showed significantly down-regulated expression levels in three treatments. According to the tissue expression pattern analysis, the expression levels of *PnCYP716A1* and *PnCYP716A6* were highest in the roots, which are the main medicinal tissue of *P. notoginseng* [2]. These two genes both responded to MEJA and ABA treatment. *PnSE2*, *PnSE3*, *PnDS1*, *PnCYP716A1* and *PnCYP716A6* showed a significant correlation of expression (Fig. 8a). *PnCYP* genes participating in the synthesis of hormones and saponin pathways were showed in Fig. 8b.

## Conclusions

We identified 188 *PnCYP* genes, which were divided into 40 subfamilies and clustered into 9 clans. We have performed phylogeny, gene structure, chromosome location, duplicated event, GO and KEGG annotation, and functional divergence analysis on this supergene family, which showed that *PnCYP* genes have undergone frequent evolution and functional diversity. Through the analysis of the transcriptome under different nitrogen treatments, some genes involved in the synthesis of plant hormones such as ABA, JA and GA were identified, and some genes involved in saponin synthesis were also identified. These five genes were screened for tissue expression pattern analysis and the expression level change analysis under hormone treatment. Our findings can provide a reference for studying the functional diversification, the supergene family members correlation, and researched the *PnCYP* genes involved in the biosynthesis of notoginseng saponins.

## Methods

### Identification and characterization of *PnCYP450s*

The *P. notoginseng* sequences were downloaded from the Chinese Herbal Plant Genome Database (http://www.herbal-genome.cn) [48] and CNGBdb Public website (http://ftp.cngb.org/pub/CNSA/). PnCYPs were identified according to the following procedure. First, we downloaded the CYP450 sequences of other species (*A. thaliana*, *O. sativa*) from the EnsemblPlants website (https://plants.ensembl.org/index.html) and *P. ginseng* CYPs from the National Center for Biotechnology Information (NCBI) website (https://pubmed.ncbi.nlm.nih.gov/30512034/). The *Populus trichocarpa* and *Physcomitrella patens* CYP450 protein sequences were downloaded from the P450 Homepage (http://drnelson.uthsc.edu/cytochromeP450.html) [32]. The AtCYP450s, OsCYP450s and PgCYP450s were compared with the *P. notoginseng* protein database by local blast comparison, and we searched *P. notoginseng* genes that had sequence similarities with those CYP450s. Next, Hidden Markov Model (HMM) profile in the Pfam database [49] (http://pfam.janelia.org/search/sequence) and NCBI Conserved Domains searches (http://www.ncbi.nlm.nih.gov/Structure/cdd/wrpsb.cgi) were peformed to see if these candidate genes contained the P450 domain (PF00067). Finally, the genes without a complete P450 domain were removed. The number of amino acids, open reading frame (ORF) length, isoelectric point (pI) and molecular weight (Mw) were estimated by ExPASy (http://www.expasy.ch/tools/pi_tool.html) [50].

### Phylogenetic tree construction and structure analysis of PnCYP450 gene family members

To investigate the phylogenetic relationships among the CYPs, the CYP450 protein sequences of *Physcomitrella patens* (moss), *O. sativa* (rice), *A. thaliana* (Arabidopsis), *P. trichocarpa* (poplar), *P. notoginseng* and *P. ginseng* (*P. ginseng*) were aligned by Clustalw. Neighbor-Joining method was employed to construct an un-rooted phylogenetic tree using the MEGA 5.0 software, and the replications were set as 1000 [51]. Both the phylogenetic trees of six species and individual *P. notoginseng* phylogenetic trees were constructed using the same method.

The Multiple Expectation Maximization for Motif Elicitation (MEME) online tool (http://meme.sdsc.edu/meme/intro.html) [52] was used to identify PnCYP conserved motifs with the following parameters: the number of different motifs was set as as 20, and the range of motif width were set as 6 to 50.

Chromosome sequence information was downloaded from NCBI website with the accession number JACBWS000000000 [53]. And chromosome location map was utilized by TBtools [54].

### Cis-regulatory elements and miRNA targets prediction

The 1500 bp upstream/downstream sequences of each *PnCYP* gene encoding region were extracted by TBtools [54]. The sequences were then submitted and analyzed on PlantCARE website (http://bioinformatics.psb.ugent.be/webtools/plantcare/html/) to identify putative cis-elements in promoter [55]. The putative cis-acting elements relative to phytohormone responses, the regulation of plant growth and development, biotic and abiotic stress responses were summarized. The *P. notoginseng* miRNA sequences were downloaded from the NCBI website (https://www.ncbi.nlm.nih.gov/pmc/articles/PMC5573331/) [56]. Targets of miRNA were identified by psRNATarget website (http://plantgrn.noble.org/v1_psRNATarget/?function=3) [57], and the parameters were set as follow: maximum expectation = 3.0, top target genes for each small RNA = 200, length for complementarity scoring (hspsize) = 20, target accessibility - allowed maximum energy to unpair the target site (UPE) = 25.

### Selective pressure analysis

Phylogeny-based and bidirectional best-hit methods were used to identify the homologous pair of putative paralogous pairs in *P. notoginseng*, orthologous pairs between *P. notoginseng* and *A. thaliana*, *P. notoginseng* and *P. ginseng* [58]. Nucleotide sequences were used in this analysis. The length of each gene in a homologous pair was longer than 300 bp, and the sequence similarity between two genes in each homologous pair was greater than 40% [59]. MEGA 5.0 was used to perform genes pair alignment, and Dnasp 5.10.1 software [60] was used to calculate the synonymous substitution rate (Ks) and non-synonymous substitution rate (Ka) substitutions per site between duplicated gene pairs [61]. Values of (Ka/Ks) < 1, = 1 and > 1 indicate negative selection, neutral evolution and positive selection, respectively [62].

### GO, KEGG annotation analysis and subcellular localization prediction

The 188 PnCYP450 protein sequences were annotated using the Blast2GO software [63, 64]. The annotation results were obtained with following parameters: e-value was 1.0E-6, annotation cutoff was 55, Go weight was 5. KAAS website (https://www.genome.jp/kaas-bin/kaas_main) and BlastKOALA website (https://www.kegg.jp/blastkoala/) were used to perform KEGG annotation [65]. On the KAAS website, the BBH (bi-directional best hit) assignment method was used. And *A.thaliana*, *P. trichocarpa* and *Vitis vinifera* were selected as GENES data set. The family_eukaryotes genus_prokaryotes database was used on the BlastKOALA website. The subcellular localization of the PnCYP proteins was predicted using CELLO v.2.5 (http://cello.life.nctu.edu.tw/) [66].

### Estimation of functional divergence

The DIVERGE 3.0 software was used to study whether functional divergence occurred among the CYPs subfamily members [67]. There were two main types of functional divergence: Type I represented the evolutionary rate change between replicated genes, where a site that was conserved in one gene but changed in another gene, and Type II represented the evolutionary nature change of replicated genes [68, 69]. If the correlation coefficients (θ_I_ and θ_II_) were significantly greater than 0, it means that functional divergence has occurred. We then calculated posterior probability value (Q_k_), and when a site with a Q_k_ value greater than 0.67, it can be defined as the functional divergence-related site. SPSS software was used for a t-value test to analyze the significant difference of the posterior probability value, and the significance threshold set as *P < 0.05.

### Expression and co-expression network and analysis of *PnCYP450* genes treated with different forms of nitrogen fertilizers

To study the expression levels of *PnCYP450* genes when *P. notoginseng* roots were treated with different forms of nitrogen fertilizers (15 mM NH^4+^ [15A], 15 mM NO^3−^ [15 N], 15 mM NH^4+^+ 15 mM NO^3−^ [15AN]), the RNA-seq data were downloaded from NCBI GEO DataSet (https://www.ncbi.nlm.nih.gov/gds/) with accession number GSE112437 and NCBI Sequence Read Archive (SRA) database (https://www.ncbi.nlm.nih.gov/sra) with accession number SRP136626. Genes with fold change (FC) greater than 1.5 and significant *p*-values < 0.05 were defined as differentially expressed genes [70]. The FPKM values of *PnCYPs* and other genes which participated in hormone synthesis were used for heat map generation by TBtools software. And scale methods were set as a row scale in the heat map drawing. Co-expression networks were performed using the OmicStudio tools (https://www.omicstudio.cn/index), and *p*-values < 0.05 were considered statistically significant.

### Plant material and real-time PCR analysis

Growing two-year-old *P. notoginseng* was collected from the Wenshan County, Yunnan Province, China. For plant hormone treatment, *P. notoginseng* leaves were treated with 10 μM ABA and 100 μM MEJA. These treated *P. notoginseng* plants were grown in greenhouses under 14/10 h of light/dark conditions at a continuous temperature of 23 ± 1 °C. Total RNA of untreated plant roots, stems, leaves flowers and hormone-treated leaves were extracted using Trizol (Sangon Biotech, Shanghai, China) according to the manufacturer’s instructions. First-strand cDNA was synthesized by a Prime-Script™ RT Reagent Kit (TaKaRa, Tokyo, Japan) according to the manufacturer’s instructions. Primer Premier 5.0 was used to design the specific primers (Table S13) required in real-time PCR experiments, 26S RNA was used as an internal reference [71]. In qRT-PCR, 20 μL reaction volume contained 10 μL 2 × *ApexHF* FS PCR Master Mix, 0.4 μL forward primer, 0.4 μL reverse primer, 1 μL cDNA template and 8.2 μL ddH2O. qPCR reaction program was set as follows: 98 °C for 10 s; 35 cycles of 55 °C for 10 s and 72 °C for 10 s. GraphPad 8.0 software was used for data analysis.

## Supplementary Information

**Additional file 1: Figure S1.** Phylogeny of CYP450s from Panax notoginseng, *Arabidopsis thaliana*, *Panax ginseng*, *Oryza sativa*, *Populus trichocarpa* and *Physcomitrella patens*.

**Additional file 2: Figure S2.** Distribution of Ka and Ks from paralogous (Pn-Pn) and orthologous (Pn-Pg and Pn-At) gene pairs. Different shapes and colors represented homologous gene pairs of Pn-Pn, Pn-Pg and Pn-At, respectively, and the black line indicates that the slope of Ka/Ks = 1.

**Additional file 3: Figure S3.** Evolutionary and gene structure analysis of PnCYP genes. Schematic representation of 20 conserved motifs in PnCYP genes. Conserved motifs in PnCYP genes were identified using MEME. Different colored boxes represent different motifs. Box lengths in the figure do not represent actual relative motif sizes. Exons and introns are indicated by yellow rectangles and gray lines, respectively.

**Additional file 4: Figure S4.** Differential expression of PnCYP genes under nitrogen fertilizers treatment. 15A:15 mM NH^4+^, 15 N:15 mM NO^3−^, 15AN:15 mM NH^4+^+ 15 mM NO^3−^

**Additional file 5: Table S1.** Detailed information about the PnCYPs in the *Panax notoginseng*.

**Additional file 6: Table S2.** Paralogous (Pn-Pn) and orthologous (Pn-Pg and Pn-At) gene pairs.

**Additional file 7: Table S3**. Ka, Ks, Ka/Ks values for the PnCYPs genes in *Panax notoginseng*.

**Additional file 8: Table S4.** Ka, Ks, Ka/Ks values for the PnCYPs genes in *Panax notoginseng*, *Panax ginseng*, and *Arabidosis*.

**Additional file 9: Table S5.** Number of each cis-acting element in the promoter regions of PnCYPs genes.

**Additional file 10: Table S6.** List of predicted miRNA-target interactions.

**Additional file 11: Table S7.** Detailed information list of GO annotation and subcellular localization prediction of PnCYP proteins.

**Additional file 12: Table S8.** List of CYP51, CYP74, CYP97 members functional divergence results.

**Additional file 13: Table S9.** List of FKPM values of 187 PnCYP450s under three kinds of nitrogen fertilizers treatment.

**Additional file 14: Table S10.** List of 107 PnCYP genes whose expression data (FPKM) > 1 in one or more treatment.

**Additional file 15: Table S11.** List of 47 PnCYP differentially expressed genes.

**Additional file 16: Table S12.** List of identified P. notoginseng ABA, JA, GA, saponins biosynthesis related genes.

**Additional file 17: Table S13.** Primers used for the qRT-PCR analysis of PnCYP gene expression.

## Data Availability

All the supporting data are included within the article and its additional files.

## Abbreviations

CYP: Cytochrome P450
GO: Gene Ontology
*P. notoginseng*: *Panax notoginseng*
AOS: Allene oxide synthase
JA: Jasmonate
ABA: Abscisic acid
ABA8ox: ABA 8′-hydroxylases
GA: Gibberellin
KO: Ent-kaurene oxidase
KAO: Ent-kaurenoic acid oxidase
BR: Brassinosteroid
HMM: Hidden Markov Model
ORF: Open reading frame
pI: Isoelectric point
Mw: Molecular weight
Arabidopsis: *Arabidopsis thaliana*
rice: *Oryza sativa*
moss: *Physcomitrella patens*
poplar: *Populus trichocarpa*
*P. ginseng*: *Panax ginseng*
MEME: Multiple Expectation Maximization for Motif Elicitation
UPE: Unpair the target site
Ks: Synonymous substitution rate
Ka: Non-synonymous substitution rate
Q_k_: Posterior probability value
15A: 15 mM NH^4+^
15N: 15 mM NO^3−^
15AN: 15 mM NH^4+^+ 15 mM NO^3−^
SRA: Sequence Read Archive
FC: Fold change
FPKM: Per Million mapped reads
SA: Salicylic acid
SS: Squalene synthase
SE: Squalene epoxidase
DS: Dammarenediol-II synthase
