# Supplementary figures and images for: Genome and transcriptome analysis to understand the role diversification of cytochrome P450 gene under excess nitrogen treatment
Source: BMC Plant Biol. 2021 Oct 6;21:447. doi: 10.1186/s12870-021-03224-x (PMC8493724; doi:10.1186/s12870-021-03224-x)

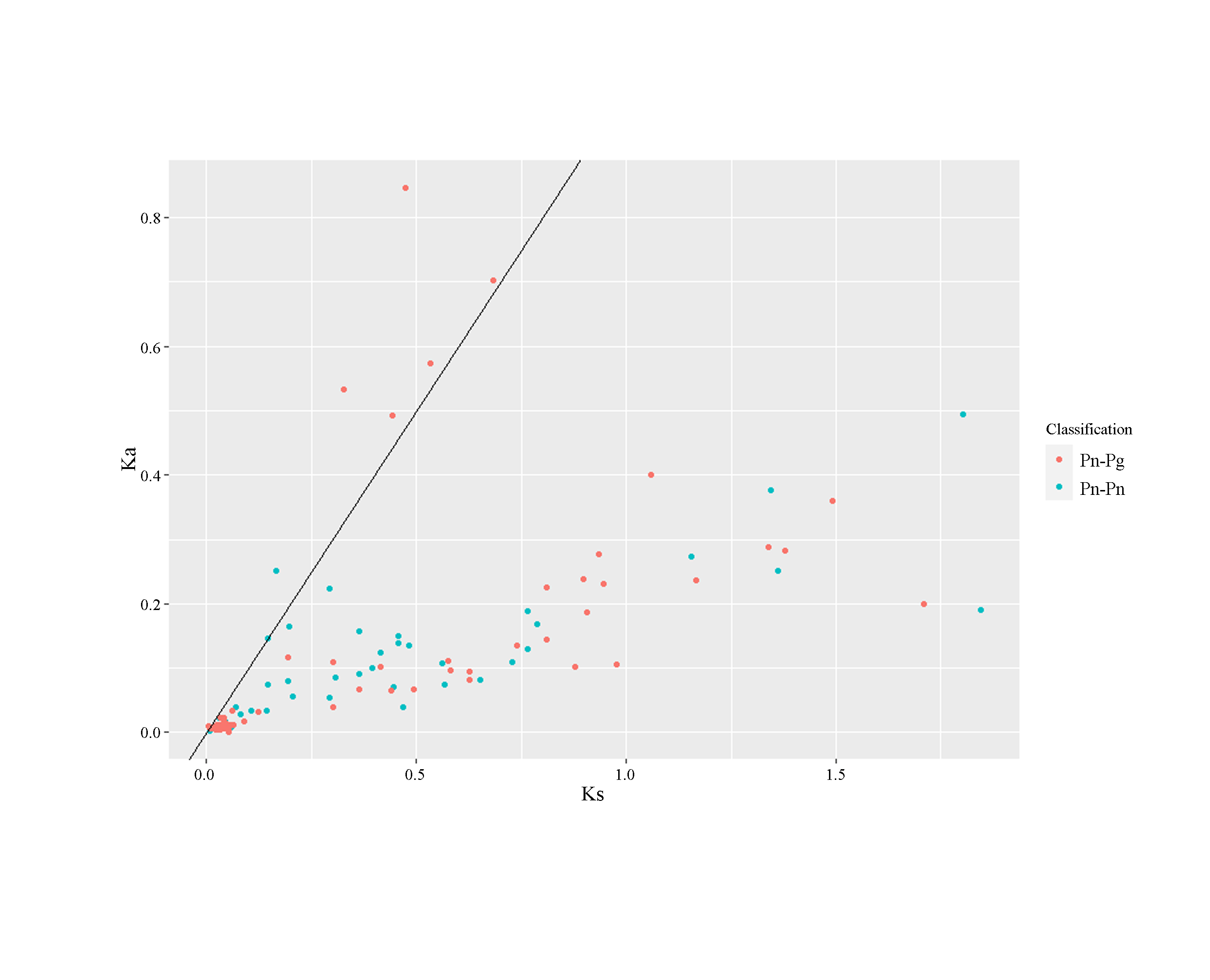

Supplement: Supplementary file 2 — Additional file 2: Figure S2. Distribution of Ka and Ks from paralogous (Pn-Pn) and orthologous (Pn-Pg and Pn-At) gene pairs. Different shapes and colors represented homologous gene pairs of Pn-Pn, Pn-Pg and Pn-At, respectively, and the black line indicates that the slope of Ka/Ks = 1. [file 12870_2021_3224_MOESM2_ESM.jpg]

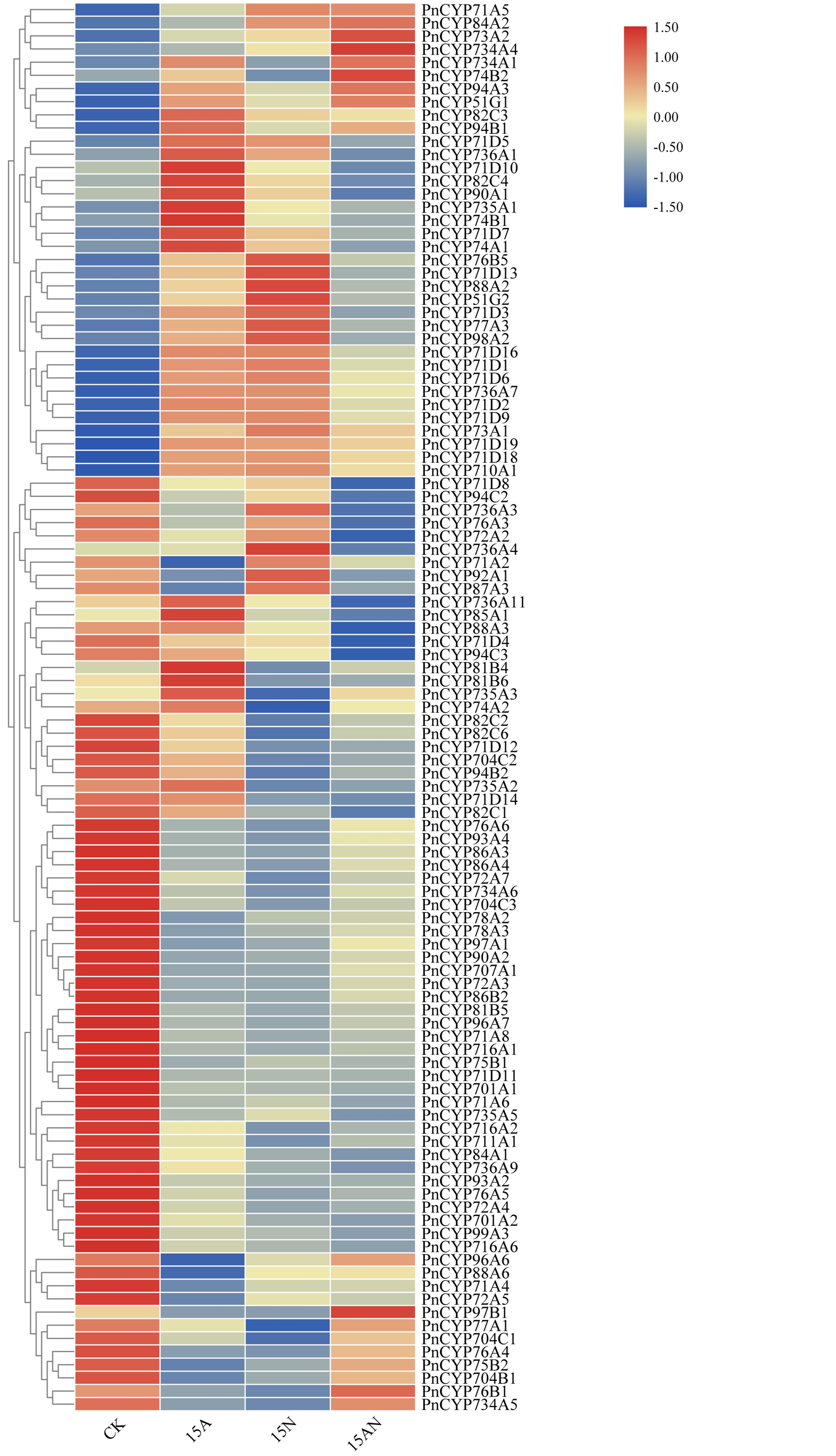

Supplement: Supplementary file 4 — Additional file 4: Figure S4. Differential expression of PnCYP genes under nitrogen fertilizers treatment. 15A:15 mM NH4+, 15 N:15 mM NO3−, 15AN:15 mM NH4++ 15 mM NO3− [file 12870_2021_3224_MOESM4_ESM.jpg]
